# Guselkumab treatment normalizes the stratum corneum ceramide profile and alleviates barrier dysfunction in psoriasis: results of a randomized controlled trial

**DOI:** 10.1016/j.jlr.2024.100591

**Published:** 2024-07-09

**Authors:** Jannik Rousel, Catherine Mergen, Menthe E. Bergmans, Lisa J. Bruijnincx, Marieke L. de Kam, Naomi B. Klarenbeek, Tessa Niemeyer-van der Kolk, Martijn B.A. van Doorn, Joke A. Bouwstra, Robert Rissmann, Next-Generation ImmunoDermatology Consortium (NGID)

**Affiliations:** 1 Centre for Human Drug Research, Leiden, The Netherlands; 2 Leiden Academic Centre for Drug Research, Leiden University, Leiden, The Netherlands; 3 Department of Dermatology, Leiden University Medical Center, Leiden, The Netherlands; 4 Department of Dermatology, Erasmus Medical Centre, Rotterdam, The Netherlands

**Keywords:** Skin, Ceramides, Lipidomics, Clinical trials, Stratum Corneum, Psoriasis, Barrier

## Abstract

The epidermal inflammation associated with psoriasis drives skin barrier perturbations. The skin barrier is primarily located in stratum corneum (SC). Its function depends on the SC lipid matrix of which ceramides constitute important components. Changes in the ceramide profile directly correlate to barrier function. In this study, we characterized the dynamics of the barrier function and ceramide profile of psoriatic skin during anti-Interleukin-23 therapy with guselkumab. We conducted a double-blind, randomized controlled trial in which 26 mild-to-severe plaque psoriasis patients were randomization 3:1–100 mg guselkumab or placebo for 16 weeks and barrier dynamics monitored throughout. Barrier function was measured by trans-epidermal water loss measurements. Untargeted ceramide profiling was performed using liquid chromatography-mass spectrometry after SC was harvested using tape-stripping. The barrier function and ceramide profile of lesional skin normalized to that of controls during treatment with guselkumab, but not placebo. This resulted in significant differences compared to placebo at the end of the treatment. Changes in the lesional ceramide profile during treatment correlated with barrier function and target lesion severity. Nonlesional skin remained similar throughout treatment. Guselkumab therapy restored the skin barrier in psoriasis. Concomitant correlations between skin barrier function, the ceramide profile, and disease severity demonstrate their interdependency.

Psoriasis is a chronic inflammatory skin disease characterized by erythematous, indurated, and scaly lesions. Psoriasis can be increasingly well-managed in clinical practice, but the presence of any residual lesions can still remain a significant burden to patients (1). While being primarily an immune-mediated inflammatory condition, cutaneous barrier perturbations allow exogeneous substances to cross the skin barrier and enhance epidermal dysregulation (2). Indeed, barrier dysfunction is observed in lesional skin and, although not observed consistently throughout all studies, also in nonlesional skin (3, 4, 5, 6).

Proper barrier function is in part regulated by the specific composition of ceramides present in the stratum corneum (SC) (7). Ceramides are incorporated in a highly organized lipid matrix together with mostly fatty acids and cholesterol (8). This matrix presents the only continuous penetration pathway for substances crossing the intact SC. The ceramide fraction is composed of many different ceramide species which can be categorized into as many as 25 different subclasses (Supplemental Fig. S1) (9). Alterations in the ceramide composition and concomitant decreases in skin barrier function have been observed in psoriasis and other inflammatory dermatological conditions (10). In atopic dermatitis (AD), these are correlated to barrier function (11, 12, 13, 14). Additionally, in vitro studies have highlighted the potential of inflammation to affect the ceramide lipid biosynthesis and thereby aggravate barrier dysfunction (15, 16). Understanding barrier dysfunction in psoriasis might provide further insights into disease and prevent the formation of new lesions (17).

Pathomechanistic insights have identified the interleukin (IL)-23 and IL-17 axis as main driver of the aberrant immunological responses in psoriasis which enabled the development of effective targeted immunosuppressing therapies (18). Guselkumab is an anti-IL23p19 monoclonal antibody that suppresses this pathway and has shown high and lasting effectivity in clinical practice (19, 20). Besides, its pronounced anti-inflammatory effect, anti-IL-23 therapy has shown to improve barrier function in a preclinical psoriasis mouse model where psoriatic lesions were induced using the toll-like receptor 7 agonist imiquimod (21).

Although studies have shown that the barrier function is decreased and the ceramide composition altered in lesional psoriasis, their longitudinal integration with measures for disease severity remains lacking and prevents the proper association of barrier dysfunction and lipid composition with disease (22, 23, 24). Therefore, we set out to investigate skin barrier dynamics in psoriasis by performing a randomized, placebo-controlled trial using guselkumab therapy in a cohort of 26 patients over 16 weeks of treatment. In-depth characterization of the ceramide profile in lesional and nonlesional skin was enabled by a comprehensive and untargeted lipidomic platform (25). Its repeated assessment and direct within-patient association to disease severity and barrier function will strongly position barrier dysfunction in psoriasis pathogenesis.

## Materials and methods

Extended methods are present in the supplementary information.

### Study design

The SKINERGY-PP study was an investigator-initiated, exploratory, single-center, double-blinded, and placebo controlled randomized trial conducted from September 2020 to January 2023 at the Centre for Human Drug Research (Leiden, the Netherlands) and registered under NCT04394936. Ethical approval was obtained from the Medische Ethische Toetsings Commissie Brabant before study conduct commenced and adhered to the Declaration of Helsinki principles. The study included plaque psoriasis patients that were not undergoing active treatment, or with appropriate wash-out, and had at least one active lesion of sufficient size to allow for sampling and with a minimal lesion severity score ≥6. The use of medication prior and during the study was prohibited, including the application of any topical products on lesions subjected to assessment. Control subjects without any dermatological conditions and otherwise healthy were included and matched for average sex, age, and tape-stripping location to the patient group. A total of 26 patients were randomized 3:1 to subcutaneous injections at week 0, 4, and 12 with guselkumab 100 mg (n = 20) or placebo (n = 6), respectively. The full in and exclusion criteria, demographics of the study population, and locations of the assessments are presented in the supplementary information.

### Clinical scoring of psoriasis severity

Severity was scored using the psoriasis area and severity index (PASI) (26). Additionally, the lesion severity score (LSS) of the target lesion was obtained by grading erythema, scaling, and induration on a 5-point scale (0; clear – 4; severe) and summing these scores (27, 28).

### Trans-epidermal water loss

Assessments were performed under controlled environmental conditions of <60% humidity and 22 ± 2°C. Cutaneous water loss was determined using an AquaFlux AF200 (Biox Systems Ltd., London, United Kingdom) over 180 s or until steady state was reached.

### Tape-stripping and ceramide lipidomics

Subsequently, a total of eight tapes (Nichiban, Tokyo, Japan) were pressed onto the skin using a D500-pressure instrument (D-squame, Cuderm Corporation, Dallas, TX) of which tape 5–8 were used for analysis. A 16 mm diameter circle was punched out and stored in chloroform:methanol (2:1) awaiting extraction. Tapes were extracted using a modified 4-step Bligh and Dyer extraction at elevated temperature as described by Boiten *et al.* (2016) (25). Extracts from tape strips 5–8 were pooled for analysis. Lipidomics was performed using liquid chromatography-mass spectrometry as described in the supplemental materials.

### Statistics

Statistical testing for the subclass profile was performed in comparison to baseline using a mixed-effect model with the Geisser-Greenhouse correction and Bonferroni correction for multiple comparisons in Prism 9.0 (GraphPad, Software, Boston). Comparisons between Guselkumab and placebo were performed within a mixed effects model with treatment and time and treatment by time as fixed factors and subject as random factor in SAS 9.4 (SAS Institute Inc., Cary, NC). Repeated measures correlation was performed using Rmcorr in R Statistical Software (version 4.1.2, R Core Team 2021) (29). (Change in) Mean and 95% confidence intervals (95%CI) are reported. Statistical significance is shown as follows: *P* ≥ 0.05: ns, *P* < 0.05: ∗, *P* < 0.01: ∗∗ and *P* < 0.001: ∗∗∗.

## Results

A total of 26 patients with plaque psoriasis and 10 heathy controls were included in the study (Supplemental Fig. S2). Demographics are listed in Supplemental Table S1 and the adverse events associated with guselkumab or placebo treatment have been reported previously (30, 31).

### Guselkumab therapy is effective and normalized the skin barrier function

**Fig. 1:**
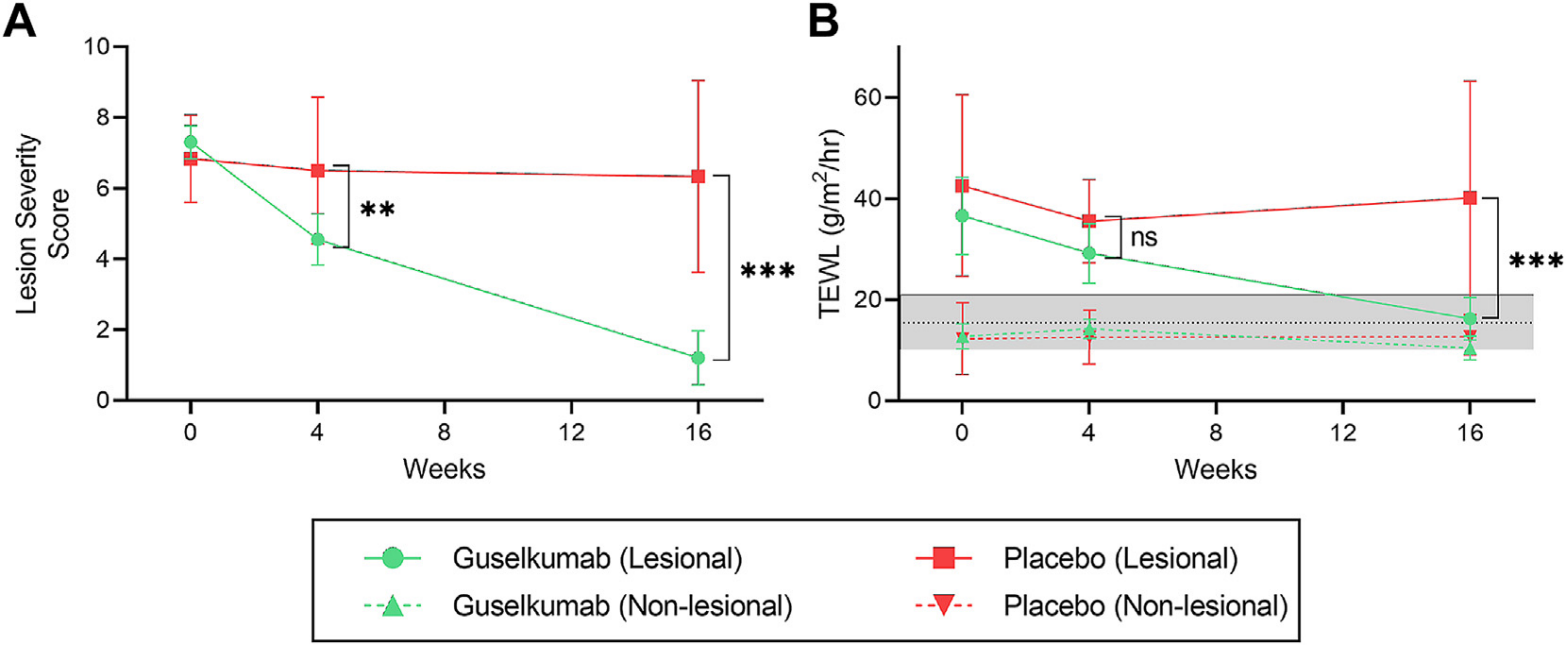
Clinical severity and barrier function during treatment with guselkumab (n = 20) or placebo (n = 6). The local severity at the target plaque subjected to assessments in this study is determined using the Lesion Severity Score (A). Barrier function as determined by trans-epidermal water loss (TEWL) was determined for both lesional and nonlesional skin (B). No significant difference was observed over time for nonlesional skin. Graphs represent the mean and 95% confidence interval. The dotted black line and gray band represent the mean and 95% confidence interval of the control group at baseline.

Effective disease remission was induced in the guselkumab group, as the plaque that was subjected to all assessments showed a significant reduction in local LSS compared to placebo throughout 16 weeks of treatment (1.20, 95%CI [0.43, 1.97] vs. 6.33 95%CI [3.62, 9.04], *P* < 0.001) (Fig. 1). In parallel, the whole-body PASI score decreased significantly compared to placebo (0.91, 95%CI [0.46, 1.36] vs. 5.86, 95%CI [12.13, −0.40], *P* < 0.001) (Supplemental Fig. S3). Complete skin clearance was not obtained since PASI and LSS scores were >0 after 16 weeks of guselkumab treatment.

Skin barrier function was measured by trans-epidermal water loss (TEWL). TEWL in lesional skin decreased significantly during guselkumab treatment compared to placebo indicating a decrease in barrier dysfunction (16.3 g/m^2^/hours, 95%CI [12.1, 20.5] vs. 40.2 g/m^2^/hours, 95%CI [17.12 63.3], *P* < 0.001). TEWL of lesional skin was comparable to that of nonlesional skin after 16 weeks of treatment (11.5 g/m^2^/hours, 95%CI [9.6, 13.3]) and controls (15.5 g/m^2^/hours, 95%CI [10.1, 21.0]). No significant changes in the TEWL of nonlesional skin over time were observed.

### The ceramide profile normalizes during treatment with guselkumab but not placebo

**Fig. 2:**
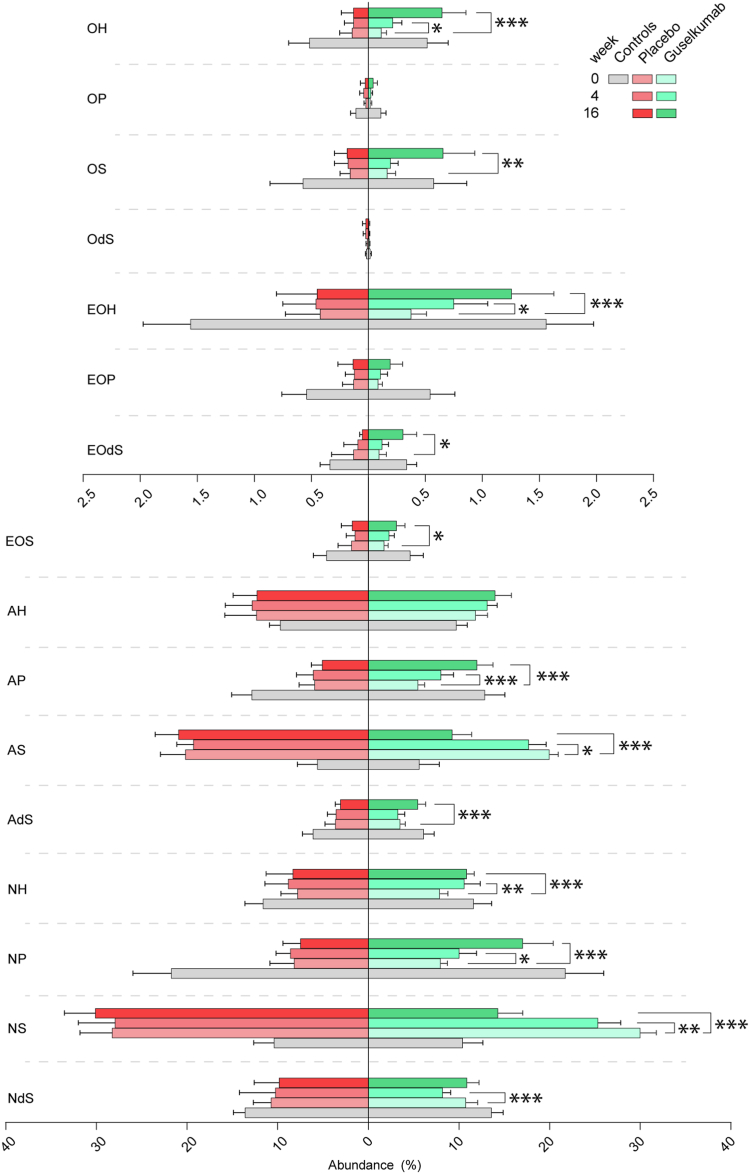
The ceramide subclass profile in lesional skin of psoriasis patients after 16 weeks of guselkumab or placebo treatment compared to controls at baseline. The ceramide subclass profile of the lesional skin of patients after 0, 4, and 16 weeks of guselkumab (n = 20) or placebo (n = 6) treatment. The ceramide profile of healthy controls (n = 10) determined during a single occasion is shown as reference. Note the different x-axis for the Cer[EO] and Cer[O] fraction because of their low abundance. Pairwise comparisons are only reported within treatment groups between baseline and week 4 or week 16.

After determining the skin barrier function, tape stripping was performed at the same site to obtain SC for analysis of the ceramide profile. No evident change in total ceramide content was observed (figure s4). However, a significant increase in the average abundance of Cer[NP] (2.059%, 95%CI [3.732, 0.3868], *P* = 0.01), Cer[NH] (2.737%, 95%CI [4.332, 1.142], *P* = 0.001), and Cer[AP] (2.524%, 95%CI [3.912, 1.136], *P* < 0.001) was observed compared to baseline already after 4 weeks of guselkumab treatment (Fig. 2). At 16 weeks, these abundances remained significantly increased (Cer[NP]; 9.074%, 95%CI [12.78, 5.366], *P* < 0.001, Cer[NH]; 2.999%, 95%CI [4.138, 1.859], *P* < 0.001, Cer[AP] 6.506%, 95%CI [8.356, 4.657], *P* < 0.001). The abundance of Cer[Ads] (1.958%, 95%CI [2.921, 0.9943], *P* < 0.001) and Cer[EOS] (1.38%, 95%CI [2.474, 0.2853], *P* = 0.01) were additionally significantly increased compared to baseline at 16 weeks. Contrarily, a significant decrease in the abundance of Cer[NS] (week 4: −4.674%, 95%CI [−8.013, −1.335], *P* = 0.006, week 16: −15.71%, 95%CI [−19.69, −11.72], *P* < 0.001) and Cer[AS] (week 4: −2.263%, 95%CI [−4.069, −0.4564,], *P* = 0.01, week 16: −10.72%, 95%CI [−13.02, −8.419], *P* < 0.001) was observed from week 4 onwards. Although Cer[NdS] initially significantly decreased at week 4 (−2.567%, 95%CI [−3.977, −1.157], *P* < 0.001), this decrease was not maintained at 16 weeks (0.1143%, 95%CI [−2.375, 2.146], *P* > 0.99). The abundance of ceramides from the Cer[O] fraction, as well as several Cer[EO] subclasses, was low in controls and was even further decreased in lesional psoriasis at baseline. Despite this, a treatment effect could be detected in the guselkumab group. The abundance of Cer[EOH] (0.3742%, 95%CI [0.7268, 0.02154], *P* = 0.04) and Cer[OH] (0.1005%, 95%CI [0.1953, 0.005718], *P* = 0.04) increased significantly after 4 weeks of treatment. Additionally, Cer[EOdS] (0.2104%, 95%CI [0.3801, 0.04069], *P* = 0.01), Cer[OS] (0.4892%, 95%CI [0.8398, 0.1387], *P* = 0.006), and Cer[EOS] (1.38%, 95%CI [2.474, 0.2853], *P* = 0.01) abundances significantly increased after 16 weeks of guselkumab treatment. No significant differences were observed in the abundances of Cer[AH], Cer[EOP], Cer[OdS], and Cer[OP] over time for the guselkumab group. In the placebo group, no significant changes compared to baseline were observed in the subclass profile at all. The actual amounts of the ceramide subclasses are shown in Supplemental Table S3.

**Fig. 3:**
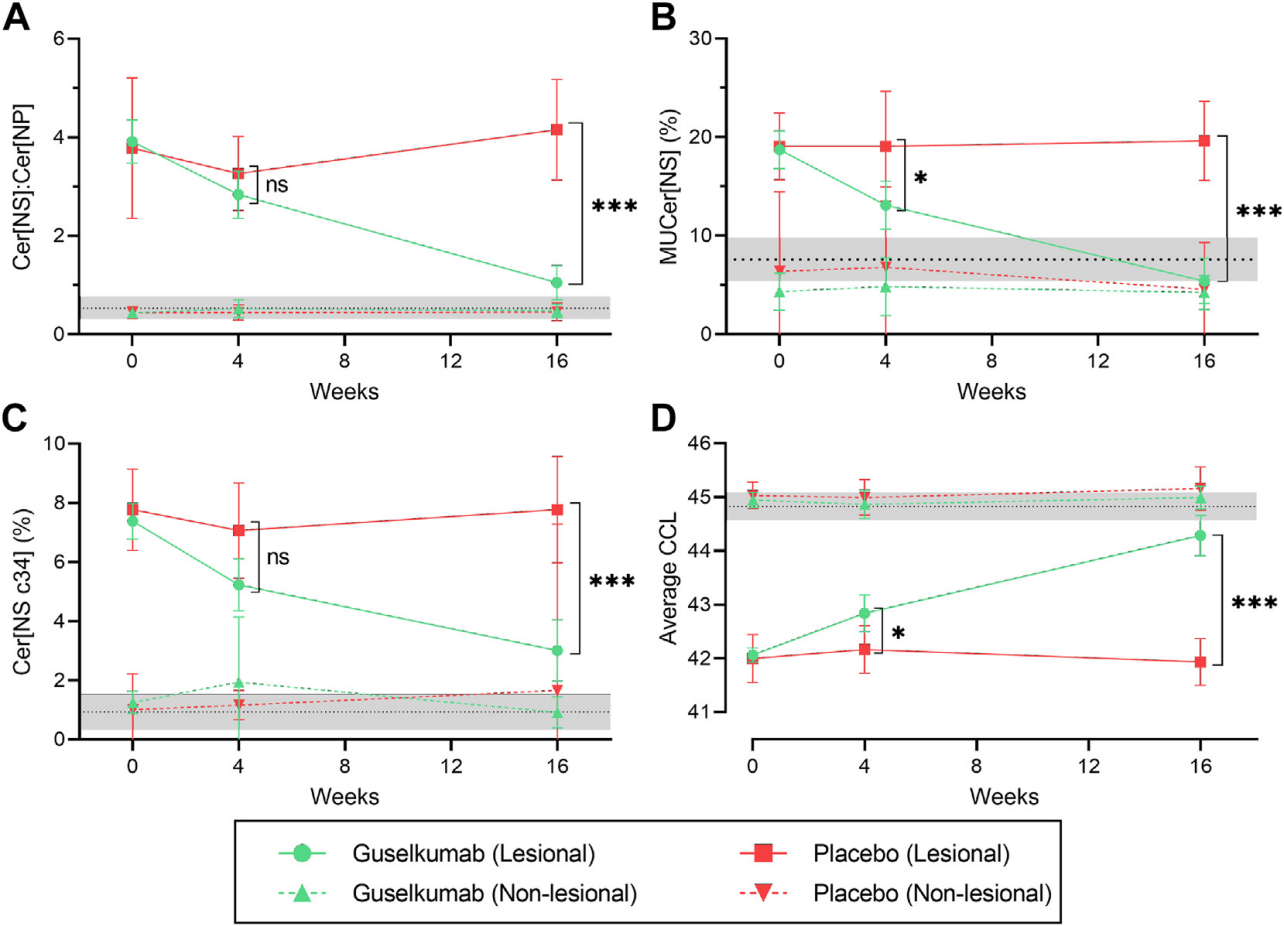
The effect of guselkumab treatment on disease severity and ceramide parameters over time in subjects randomized to guselkumab (n = 20) or placebo (n = 6). Lesional and nonlesional skin is followed over time with ceramide profiling yielding the Cer[NS]:Cer[NP] ratio (A), percentage monounsaturated Cer[NS] (MUCer[NS]) of total Cer[NS] (B), percentage Cer[NSc34] of total Cer[NS] (C), and the average ceramide chain length (CCL) of the Cer[A] and Cer[N] fraction (D) as representative parameters of the altered ceramide profile at baseline. Graphs represent the mean and 95% confidence interval. The dotted black line and gray band represents the mean and 95% confidence interval of the control group at baseline. *P*-values are indicated when comparing lesional skin of the guselkumab group with the lesional skin of the placebo group. No statistically significant changes were present when comparing nonlesional skin in the placebo group with nonlesional skin in the guselkumab group.

In line with changes to the overall ceramide profile, specific ceramide parameters showed concurrent changes (Fig. 3). Increased abundances of Cer[NP] compared to Cer[NS] in the ceramide profile were reiterated based on a decreasing Cer[NS]:Cer[NP] ratio over time, which resulted in a significant decrease compared to placebo after 16 weeks of treatment (1.05, 95%CI [0.70, 1.93] vs. 4.15, 95%CI [3.13, 5.18], *P* < 0.001).

As Cer[NS] remains abundant in lesional and nonlesional skin, the degree of monounsaturated ceramides can be reliably detected without confounding resulting from decreases in overall Cer[NS] abundancies (32). Therefore, the percentage monounsaturated Cer[NS] (MUCer[NS]) of Cer[NS] was used as an indicator of the degree of unsaturation. The fraction MUCer[NS] decreased significantly in the guselkumab group compared to placebo from week 4 onwards (5.40%, 95%CI [3.09, 7.71], vs. 19.61%, 95%CI [15.59, 23.63], *P* < 0.05) and ultimately reached the level observed in controls after 16 weeks (guselkumab: 5.40%, 95%CI [3.09, 7.71], placebo: 19.61%, 95%CI [15.59, 23.63], *P* < 0.001, controls: 7.6%, 95%CI [5.4, 9.8]).

Constituting a very-short chain ceramide, the abundance of ceramide Cer[NS] with 34 total carbons (Cer[NSc34]) decreased over time in the guselkumab group and reached statistical significant compared to placebo after 16 weeks of treatment (3.00%, 95%CI [1.96, 4.05] vs. 7.77%, 95%CI [5.97, 9.57], *P* < 0.001). However, its abundance at 16 weeks remained markedly higher than nonlesional (1.07%, 95%CI [0.59, 1.55]) and control skin (0.53%, 95%CI [0.31, 0.77]).

Lastly, ceramide elongation was investigated. The detection of the individual and intact ceramides enables the determination of the ceramide chain length (CCL), defined as the total number of carbons within the molecule. The average CCL of Cer[N] and Cer[A] (Cer[N,A]) showed a gradual increase throughout treatment with guselkumab, but not placebo. This increase was significant compared to placebo at both week 4 (42.84 carbons, 95%CI [42.49, 43.18] vs. 42.17 carbons, 95%CI [41.72, 42.61], *P* < 0.05) and week 16 (44.28 carbons, 95%CI [43.90, 44.66] vs. 41.93 carbons, 95%CI [41.50, 42.37], *P* < 0.001). Compared to the Cer[N,A] fraction, the CCL in the Cer[EO] fraction showed a smaller but still significant increase compared to placebo after 16 weeks of treatment (68.51 carbons, 95%CI [68.29, 68.73] vs. 67.92 carbons, 95%CI [67.52, 68.31], *P* < 0.05). No significant differences were observed compared to placebo for the CCL of Cer[O] at week 4 (49.83 carbons, 95%CI [49.61, 50.14] vs. 50.00 carbons, 95%CI [49.71, 50.29], *P* = 0.87) or week 16 (49.79 carbons, 95%CI [59.49, 50.08] vs. 49.91 carbons, 95%CI [49.31, 50.52], *P* = 0.46) (Supplemental Fig. S5). Of note, results were maintained when ceramides of CCL ≤34 were excluded from the analysis (data not shown). When evaluating the aforementioned parameters in nonlesional skin, these remained consistent over time and were comparable to those observed in controls.

**Fig. 4:**
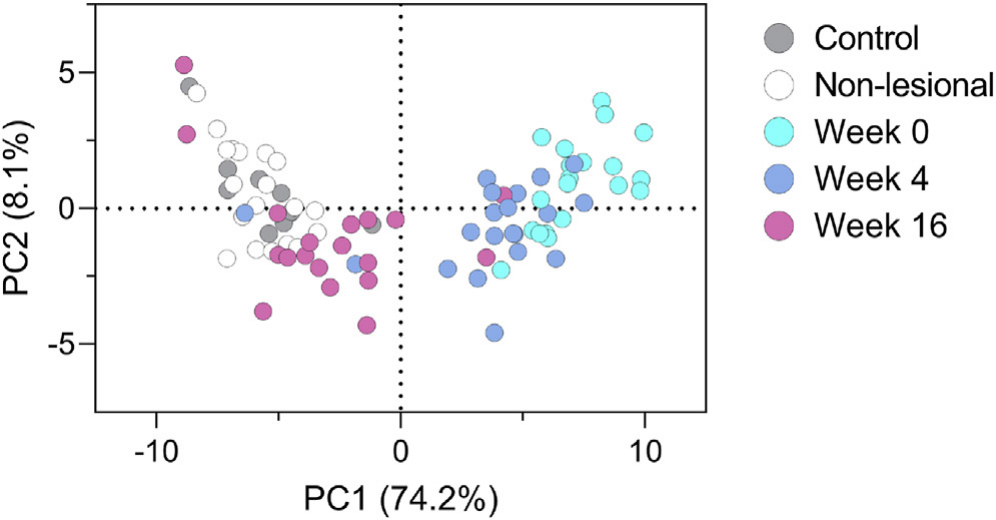
The ceramide profile of lesional skin during guselkumab treatment and the ceramide profile of nonlesional skin and healthy controls at baseline visualized by principal component analysis. The relative composition of all detected saturated ceramides is used for each data point in the PCA. Profiles of healthy controls are added as a reference for a normal ceramide profile. Additionally, the nonlesional skin of psoriasis patients at baseline (week 0) is included. A high proximity between data points indicates a high degree of similarity. The amount of variance explained per principal component (PC) is displayed on the axis.

Integration of the complete saturated ceramide profile using dimension reduction analysis visualizes the conformity between different samples based on their proximity to each other. At baseline, samples of nonlesional skin of patients are overlapping with that of controls indicating similarity. However, the ceramide profile of lesional skin appears markedly different from both control skin and nonlesional skin (Fig. 4). The lesional profile starts shifting towards that of healthy volunteers already after 4 weeks of treatment. After 16 weeks, all lesional samples have become separated from those obtained at baseline and overlap fully with those from healthy controls and nonlesional skin, indicating a high degree of similarity between these samples. Of note, two samples obtained at week 16 remain closer to those of week 4 and 0 which indicates incomplete normalization. Listing of the loadings that drive the principal component analysis indicates that the abundance of Cer[NS], Cer[AS], and Cer[NP] with CCLs between 42 to 48 carbons, and to a lesser extent those with 34 carbons, are the driving factors behind the distribution of datapoints (Supplemental Table S4).

### Ceramide parameters correlate with barrier dysfunction and disease severity

**Fig. 5:**
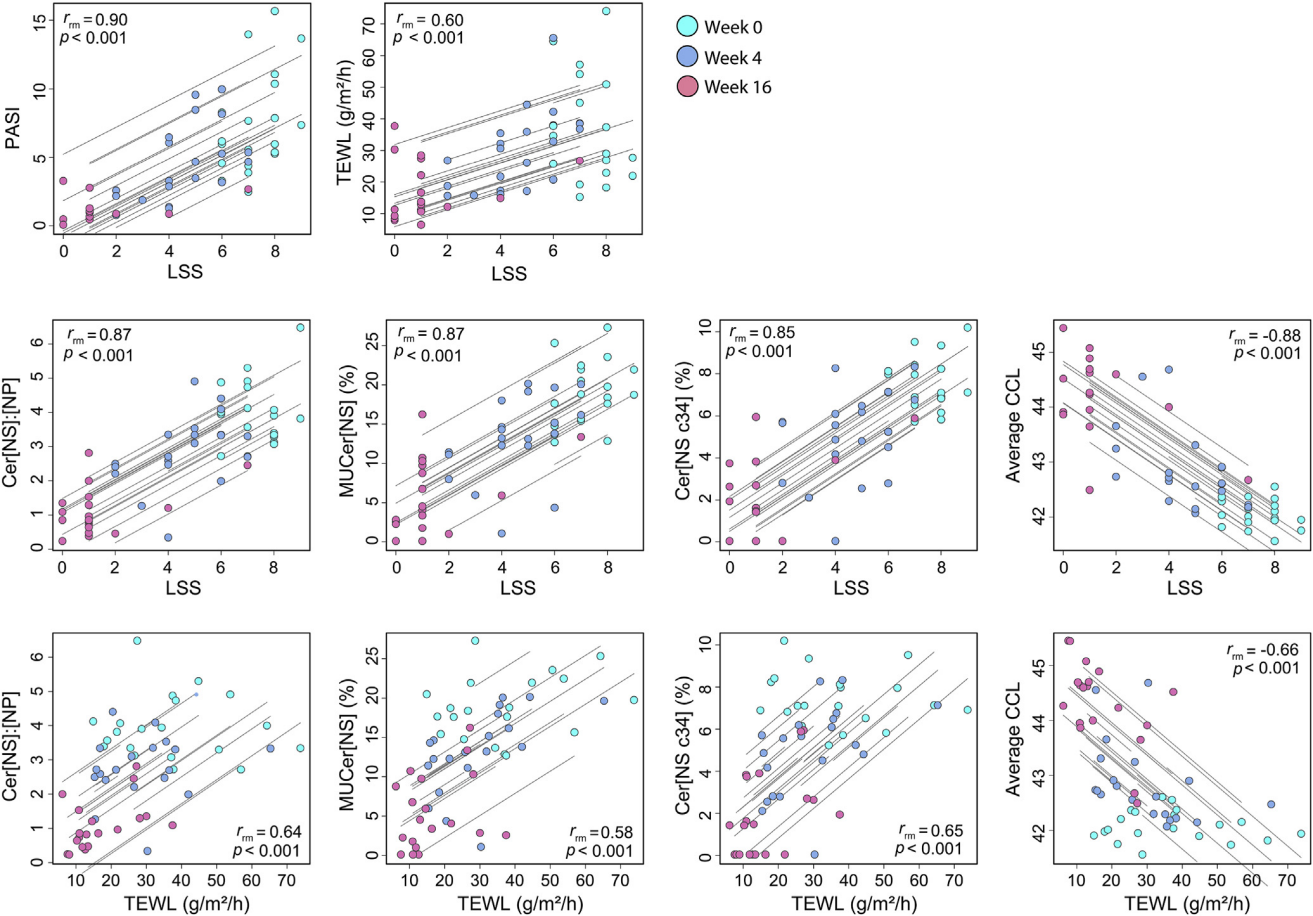
Correlation between disease severity and barrier function or ceramide parameters from patients receiving guselkumab treatment. Repeated measures correlation coefficients (r_rm_) between local lesion severity (LSS) score, the psoriasis area and severity index (PASI) and trans-epidermal water loss (TEWL) are plotted. Additionally, the Cer[NS]:Cer[NP] ratio, monounsaturated Cer[NS] (MUCer[NS]), percentage Cer[NSc34], and the average ceramide chain length (CCL) of Cer[A] and Cer[N] are contrasted with the LSS. Lastly, these ceramide parameters are correlated with TEWL. Lines indicate the common within-individual association per subject and directly relate to the r_rm_ coefficient.

The repeated assessment of disease severity, barrier function, and the ceramide profile during guselkumab treatment allows the establishment of a relationship between these parameters (Fig. 5). However, the repeated assessment of the same patient during the trial results in dependent data over time, which disqualifies the use of standard correlation analysis as this may lead to bias or wrongful results. Instead, repeated measures correlations are used as an alternative which is compatible with this dataset to determine the common within-individual association (29). Firstly, the local LSS and total-body PASI correlated strongly which indicates that both reflect the treatment response similarly (r_rm_ = 0.90). The relationship between LSS and the ceramide profile was strong with high correlation coefficients between LSS and the Cer[NS]:Cer[NP] ratio (r_rm_ = 0.87), abundance MUCer[NS] (r_rm_ = 0.87), Cer[NSc34] (r_rm_ = 0.85) and average CCL in Cer[N,A] (r_rm_ = −0.88). A significant correlation between barrier function measured by TEWL and LSS was observed (r_rm_ = 0.60). Additionally, barrier function correlated with the ceramide parameters as significant repeated measures correlations were observed between TEWL and Cer[NS]:Cer[NP] ratio (r_rm_ = 0.64), abundance MUCer[NS] (r_rm_ = 0.58), Cer[NSc34] (r_rm_ = 0.65) and average CCL in Cer[N,A] (r_rm_ = −0.66).

## Discussion

Here, we demonstrate the dynamics of barrier dysfunction and the ceramide profile as investigated during a prospective, randomized, placebo-controlled trial in psoriasis patients. Guselkumab treatment, but not placebo, normalized the full ceramide profile in lesional skin compared to that of controls thereby resolving barrier dysfunction. We show that changes in the ceramide profile correlate with both disease severity and barrier function.

### Normalization of the ceramide profile during guselkumab treatment

The barrier function of the SC is heavily dependent on the ceramides of the extracellular lipid matrix (8). The ceramide profile of lesional psoriasis is altered with marked decreases in Cer[P] abundances and a lower CCL (23, 33, 34, 35). Additionally, the abundance of ceramides with a total chain length of 34 carbons is increased (23), as also observed in this study through higher Cer[NSc34] abundances. In this study, we demonstrate the normalization of the entire ceramide profile during treatment, including the increased abundance of MUCer[NS]. Increased unsaturation had been observed in Netherton syndrome, atopic dermatitis, and seborrheic dermatitis but had not yet been described in psoriasis (32, 36, 37).

Normalization of the balance between dihydroceramide desaturase (DES)−1 and −2 activity might explain the restored Cer[NS]:Cer[NP] ratio as they mediate the final step in Cer[S] and Cer[P] synthesis, respectively (38). DES-2 has shown to be downregulated in the SC of lesional AD (39). However, abundances of Cer[dS] and Cer[H] appear altered as well. This indicates a more upstream inhibition of ceramide synthesis as Cer[dS] is the precursor for Cer[P] (38). Indeed, a DES-2 KO model indicated other enzymes might differentially contribute to Cer[P] synthesis as Cer[P] was still present despite knocking out DES-2 (40). Rather, restoration of the impaired ceramide synthesis might relate to the restoration of proper epidermal homeostasis during treatment as skewing of the Cer[NS]:Cer[NP] ratio has been related to the aberrant overall epidermal hyperplasia typical of psoriasis (41). Increased unsaturation might result from an increase in stearoyl-CoA-9-desaturase-1 activity, which mediates the rate-limiting step in monounsaturated fatty acid synthesis and therefore also monounsaturated ceramides (38, 42). The involvement of stearoyl-CoA-9-desaturase-1 in psoriasis has not been properly elucidated, but its expression was increased in AD (43). However, steoryl-CoA-9-desaturase’s upstream activators such as the liver-X receptor-alpha and sterol regulatory element binding protein-1 have previously been reported to be downregulated in lesional psoriasis instead, highlighting the need to specifically determine the expression or activity of stearoyl-CoA-9-desaturase-1 in psoriasis (44, 45).

Besides normalization of the subclass profile of lesional skin, impaired ceramide elongation is alleviated by evident increases in the CCL of Cer[N,A] and a more limited increase in the CCL of the Cer[EO] fraction. A psoriasis mouse model showed decreases in the expression of fatty acid elongases ELOVL1, ELOVL3, and ELOVL4 as well as ceramide synthase 3, which are all involved in the synthesis of ceramides exceeding 24 carbons (22). These findings are supported by studies in AD where comparable changes were observed in patients and after inducing inflammation in models (43, 46, 47). This reduction in CCL can be attributed to a decrease in the length of either the acyl chain, the sphingoid base, or both, as the length of both chains has been shown to be reduced in psoriasis (22, 48). Of note, the presence of short-chain Cer[NSc34] is increased in lesional skin as previously reported in AD, ichthyosis, and psoriasis (8, 10). These short-chain ceramides are involved in cellular stress responses and have been associated with inflammation and higher cardiovascular risk (49, 50). Their increased abundance in psoriatic skin, and subsequent decrease during guselkumab treatment, might therefore more aptly reflect the dysregulated homeostasis instead of changes to lipid elongation. However, normalization of lipid elongation might have further facilitated the decrease of Cer[NSc34] by effectively reducing the abundance of shorter fatty acids used as substrate in ceramide synthesis (38). This might differentially contribute to the decrease in cellular stress responses caused by this bioactive ceramide and aid in the normalization of ceramide synthesis altogether (51). The noninflammatory phenotype of uninvolved psoriasis is reiterated by a high similarity to healthy control skin as shown in this study (33, 52). This differentiates psoriasis from AD were nonlesional barrier defects are cemented in its pathogenesis (53). Ultimately, anti-IL-23 therapy is able to normalize the alterations in the SC ceramide profile which are associated with psoriasis and other dermatoses (10).

### Barrier restoration during guselkumab treatment

Barrier dysfunction is evidently tied to lesional psoriasis (24, 54, 55, 56). Previous studies have demonstrated that TEWL of lesional skin improves after treatment in line with clinical scores but did not monitor TEWL during treatment and correlate this directly to (local) disease severity (6, 57, 58). Here, guselkumab treatment effectively restores the barrier function of lesional skin in parallel with the clinical remission of disease. We expand on this by using a highly effective treatment contrasted with a placebo group and by performing repeated measures correlation analysis to further link disease severity to barrier dysfunction in lesional skin. TEWL values of nonlesional skin remained comparable with that of controls throughout the 16-weeks study indicating barrier impairment is limited to lesional skin, in contrast to what was observed in a smaller cross-sectional study (5).

### The effect of guselkumab on barrier function and the ceramide profile

Guselkumab prevents the association of IL-23 with its receptor, preventing the activation of IL-17 producing cells. Significant decreases in clinical disease severity compared to placebo were expected based on its efficacy in phase III trials and real-world evidence (19, 20, 59). However, little is known about the direct impact of IL-23 on ceramide synthesis. IL-23 expression by keratinocytes has shown to result in enhanced interferon-gamma responses (60). This cytokine can directly impact SC ceramide synthesis with a pronounced effect on CCL (15, 16, 61). Additionally, induction of psoriatic lesions in mice using imiquimod has shown to result in barrier dysfunction which could be effectively reduced with anti-IL-12/23 therapy (21). Although imiquimod application has also shown to alter the ceramide profile, the potential to reverse these imiquimod-induced alterations with anti-IL-23 therapy has not yet been investigated (22). Conversely, it has been shown that knocking-out serine palmitoyltransferase in mice, causing a severe impairment of ceramide synthesis leading to a reduction in overall epidermal ceramides, spontaneously develop psoriatic-like lesions with increased TEWL which was treatable with IL-12/23 therapy (62). Tumor necrosis factor represents another, more upstream, therapeutic target for psoriasis (63). Supplementation of tumor necrosis factor resulted in alterations in the ceramide profile and expression of ceramide synthesis proteins of in vivo skin models (16, 64). This preclinical basis affirms the potential of guselkumab to influence barrier dysfunction through its upstream and downstream pro-inflammatory mediators as observed in this study.

### Correlations

We performed a double-blind, randomized, placebo-controlled trial to solidly affirm the relationship between alterations in the ceramide profile, barrier dysfunction, and psoriasis. Previously, a smaller cross-sectional study linked barrier dysfunction to skewing of subclass ratios but did not consider disease severity (41). Another study showed barrier impairment with concomitant alterations in the ceramide profile but did not evaluate direct, intra-patient correlations (24). We demonstrate that alterations in the ceramide composition, unsaturation, and CCL of lesional skin are paired with decreased barrier function throughout lesions with variable severity. Correlations between changes in ceramide composition and permeability have been demonstrated using model systems that mimic the lipid matrix of the inflamed skin where a decrease in barrier function was observed by skewing Cer[NS]:Cer[NP] (65, 66), decreasing CCL (67, 68), and increasing unsaturation (69). Moreover, the altered ceramide profile is implicated in the barrier impairment of in vitro-cultured human skin equivalents (70, 71). In AD and seborrheic dermatitis, alterations in the ceramide profile have provided a basis for the observed barrier dysfunction (32, 41, 72, 73, 74).

While the repeated measure correlation indicates a relationship between barrier function and ceramide profile, the correlation between LSS and the ceramide profile is stronger. Although the skin barrier is highly dependent on the ceramide profile, other factors might differentially contribute to skin barrier dysfunction in psoriasis (17). A stronger relationship between the ceramide profile and LSS might be explained by the fact that both reflect the extent of epidermal dysregulation which as this is directly assessed by clinical scoring but can also be linked to the ceramide profile (10, 41). Of note, the association between local severity and barrier parameters was affirmed by two patients who showed incomplete local remission despite a decrease in PASI score and maintained a ceramide profile closer to baseline despite receiving guselkumab. Therefore, we focused on the LSS rather than full body PASI to reflect local disease severity more accurately. However, correlations between PASI and LSS remained high indicating that both may be used.

### Ceramide profiling as a biomarker for treatment monitoring

The relationship between treatment effect and ceramide profiles might allow for its exploitation as a biomarker. For instance, Cer[NSc34] constitutes a single biomarker that is able to discern lesional from nonlesional skin and gradually decreases during treatment. Traditional clinical endpoints, such as the observer-based physician global assessment and PASI scores, have become the gold standard but are associated with poor sensitivity, bias, and high interrater variability (26, 75, 76). Additionally, it remains a challenge to use traditional clinical endpoints in the scoring of mild psoriasis as also included in this study due to insensitivity (77, 78). Expanding clinical endpoints with objective measures might increase the value of these results (79).

Earlier, analysis of a limited amount of ceramides showed that increased Cer[NS] and decreased Cer[EOS] abundances in the skin of patients with AD normalized towards the level of controls during dupilumab treatment (80, 81). Here, we show the same effect in psoriasis during effective treatment but extent it to the entire ceramide profile. Considering the similarity of the ceramide profile in psoriasis and AD, together with its comparable response to effective treatment, ceramide profiling might also be applied to other indications where similar alterations are observed such as ichthyosis (10). However, treatment responses of plaques within the patient might differ which should be considered when only selected lesions are monitored (82). Although TEWL and ceramides are both markers of skin barrier function, ceramide readouts from lesional skin seem less variable. TEWL can be impacted by personal factors, environmental factors, and differ between measurement locations (83, 84). Although these factors can also impact the ceramide profile, their influences might be more limited (85, 86, 87, 88, 89). Results obtained with tape stripping might vary depending on sampling depth as total ceramide abundances have been shown to be higher in deeper layers of the SC (90). This might be especially relevant in psoriasis considering the presence of hyperkeratosis with excessive scaling. However, the relative ceramide subclass composition has been reported to be comparable at varying SC depths up to 33 consecutive tape strips, indicating that compositional changes might be interpreted irrespective of sampling depth (25). Tape-stripping has already been applied to obtain protein and transcriptome-based biomarkers that are able to reflect the specific inflammatory component of specific diseases 91, 92, 93. In contrast to proteins, ceramides represent less proximal biomarkers that might be more primarily related to the general inflammatory status of the skin and the resulting aberrant epidermal differentiation (41). Although this disconnect might challenge its use as a diagnostic biomarker, the more general relationship between ceramides and disease provides a more holistic view of the response to treatment. Additionally, it appears that ceramide profiling is able to detect super responders, patients with a strong or early response to treatment (94), as two subjects show a normalized ceramide profile already after 4 weeks. The ability to define a strong response through ceramide profiling might therefore highlight ceramide profiling as a suitable alternative that may be employed to predict the long-term efficacy of a chosen therapy and enable a precision medicine approach (95).

## Conclusion

In this study, we show that guselkumab therapy normalizes the ceramide profile and alleviates barrier dysfunction in psoriasis patients. Correlations between the ceramide profile and barrier function during the effective clinical response toward guselkumab, but not placebo, underline their interdependency and establish their relationship with disease severity. Ceramide profiling might represent a suitable biomarker for treatment monitoring. Variation in the study is controlled by matching the controls with patients for approximate age, sex, assessment location, and recruitment throughout seasons. Robustness of the results is underlined by low variation in the placebo group over the 16-weeks treatment period.

## Data Availability

The data that support the findings of this study are available from the corresponding author upon reasonable request.

## Supplemental data

This article contains supplemental data (12, 25, 29, 35).

## Conflict of interest

Dr van Doorn has received consulting fees or honorarium from Novartis, AbbVie, Pfizer, LEO pharma, Sanofi, Lilly, Janssen, and Celgene and grant and payment for lectures including service on speakers bureaus from Novartis, Sanofi, and Janssen outside the submitted work. The other authors declare that they have no conflicts of interest with the contents of this article.
