# Supplementary material for: Guselkumab treatment normalizes the stratum corneum ceramide profile and alleviates barrier dysfunction in psoriasis: results of a randomized controlled trial
Source: J Lipid Res. 2024 Jul 9;65(8):100591. doi: 10.1016/j.jlr.2024.100591 (PMC11342092; doi:10.1016/j.jlr.2024.100591)
Supplement: Supplemental Data [file mmc1.docx]

Supplemental information pertaining to “Guselkumab treatment normalizes the altered stratum corneum ceramide profile and alleviates barrier dysfunction in psoriasis: results of a randomized, placebo-controlled trial”

By Rousel, Mergen *et al.*


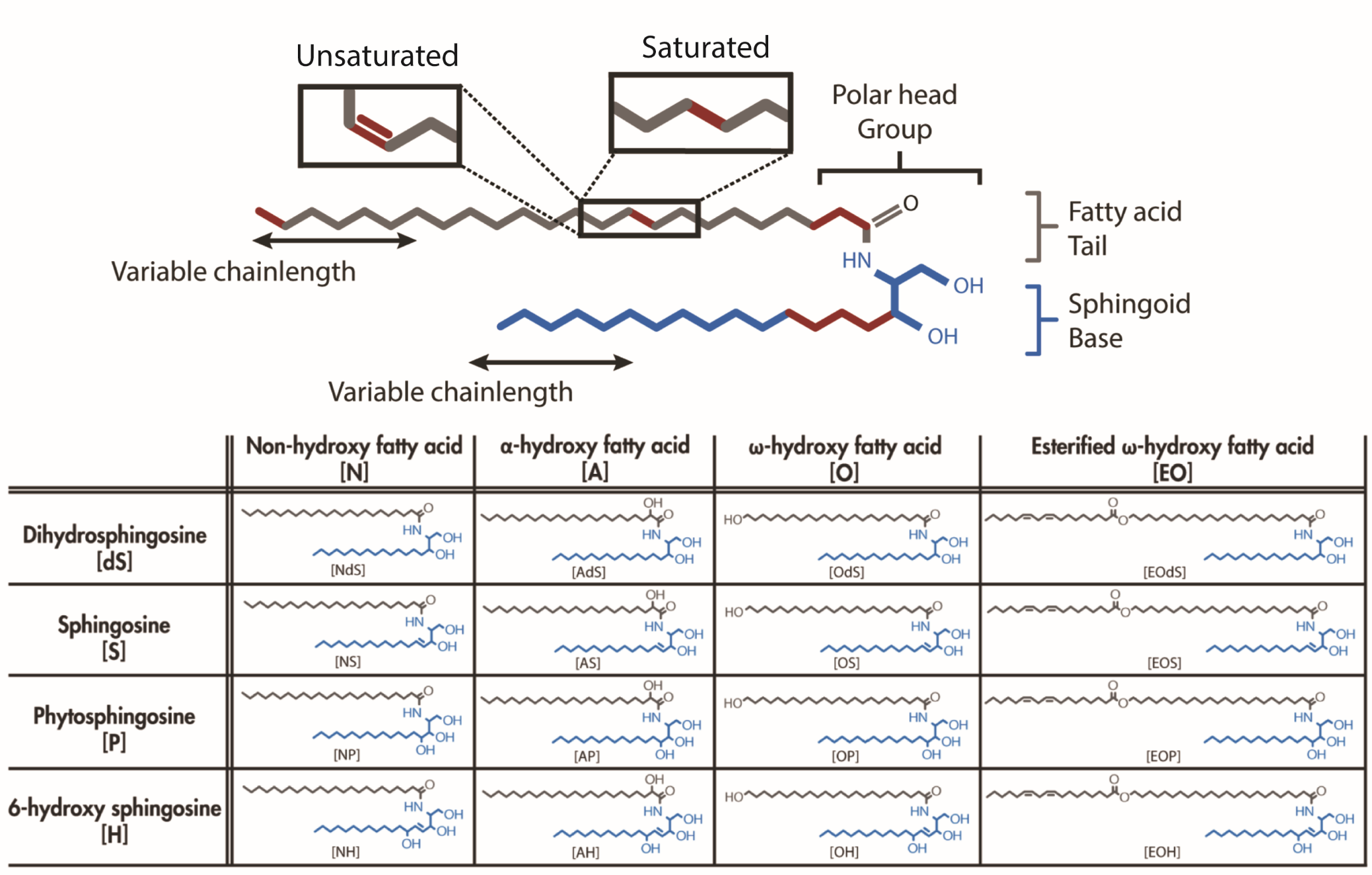


**Figure s1:** An overview of the variety in free ceramides. Adapted from Janssens *et al.* (2012).^1^ A general structure with the structural differences highlighted such as differences in headgroup architecture, chain length and degree of unsaturation. Additionally, it presents the structure of all ceramide classes used in this study according to the nomenclature by Motta *et al.* (1993).^2^

**Figure s2:** Consort flow chart of the study population.

**Table s1:** Baseline demographics. Note that locations for the assessments are provided in the supplemental Excel file containing the raw data. The location for the control group is chosen to reflect the lesional location selected for patients.

|  |  | **Controls** | **Placebo** | **Guselkumab** |
| --- | --- | --- | --- | --- |
| Total number of patients | | 10 | 6 | 20 |
| Age at first dose | ≤ 18 years | 0 (0%) | 0 (0%) | 0 (0%) |
|  | 18 - 65 years | 10 (100%) | 6 (100%) | 19 (95%) |
|  | ≥ 65 years | 0 (0%) | 0 (0%) | 1 (5%) |
|  | Average age (years±SD) | 42.6±9.8 | 44.4±12.5 | 40.2±6.9 |
| Sex | Female | 7 (70%) | 1 (17%) | 5 (25%) |
|  | Male | 3 (30%) | 5 (83%) | 15 (75%) |
| Race | White | 9 (90%) | 5 (83%) | 17 (85%) |
|  | Asian | 0 (0%) | 1 (17%) | 0 (0%) |
|  | Hispanic | 1 (10%) | 0 (0%) | 1 (5%) |
|  | More than one | 0 (0%) | 0 (0%) | 2 (10%) |
| Fitzpatrick | I | 0 (0%) | 0 (0%) | 1 (5%) |
|  | II | 5 (50%) | 3 (50%) | 8 (40%) |
|  | III | 4 (40%) | 2 (33%) | 10 (50%) |
|  | IV | 1 (10%) | 0 (0%) | 0 (0%) |
|  | V | 0 (0%) | 0 (0%) | 0 (0%) |
|  | VI | 0 (0%) | 1 (17%) | 1 (5%) |
| Psoriasis Area and Severity Score (PASI) | | - | 3.7±0.8 | 4.6±1.9 |
| Lesion Severity Score (LSS) | | - | 7.3±1.0 | 6.8±1.2 |
| TEWL (g/m^2^/h) | Lesional | 15.5±7.6 | 42.6±17.1 | 36.7±16.3 |
|  | Non-lesional | - | 12.3±6.8 | 12.8±5.4 |

**Overview of the in- and exclusion criteria for this study**

Eligible healthy controls must meet all of the following inclusion criteria at screening:

1. Male or non-pregnant female subjects, 18 to 75 years of age (inclusive); during COVID-19 pandemic this is set to 18 to 69 year of age (inclusive)
2. Healthy as defined by the absence of any uncontrolled active or uncontrolled chronic disease following a medical and surgical history, documentation of general symptoms, and a symptom-directed physical examination including vital signs;
3. Willing to give written informed consent and willing and able to comply with the study protocol;

And eligible healthy controls must meet none of the following exclusion criteria at screening:

1. History or symptoms of any uncontrolled, significant disease including (but not limited to), neurological, psychiatric, endocrine, cardiovascular, respiratory, gastrointestinal, hepatic, or renal disorder that may interfere with the study objectives, in the opinion of the Investigator;
2. History of immunological abnormality (e.g., immune suppression, severe allergy or anaphylaxis) that may interfere with study objectives, in the opinion of the Investigator;
3. Known infection requiring antibiotic therapy within the last three months prior to the study;
4. Immunosuppressive or immunomodulatory treatment within 30 days prior to the study;
5. Body mass index (BMI) ≤ 18.0 or ≥ 40.0 kg/m^2^; during COVID-19 pandemic only ≤ 18.0 or > 33.0 kg/m^2^
6. Participation in an investigational drug study within 3 months prior to screening or more than 4 times a year;
7. Previous participation in an investigational drug study involving the dosing of an investigational compound targeting an immune pathway within one year prior to screening;
8. Loss or donation of blood over 500 mL within three months prior to screening;
9. The use of any medication or vitamin/mineral/herbal/dietary supplement within less than 5 half-lives prior to study participation, if the Investigator judges that it may interfere with the study objectives. The use of paracetamol (up to 4 g/day) is allowed;
10. History of alcohol consumption exceeding 5 standard drinks per day on average within 3 months of screening. Alcohol consumption will be prohibited from at least 12 hours preceding each study visit;
11. Any other condition that could interfere with the conduct of the study or the study objectives, in the opinion of the Investigator.
12. During COVID-19 pandemic: presence of high risk comorbidities: such as cardiovascular, respiratory or immune system disorders

Eligible psoriasis patients must meet all of the following inclusion criteria at screening:

1. Male or non-pregnant female subjects, 18 to 75 years of age (inclusive); during COVID-19 pandemic this is set to 18 to 69 year of age (inclusive)
2. Diagnosed with plaque psoriasis at least 6 months prior to study participation
3. Willing to discontinue any psoriasis therapy other than emollients.
4. Having mild (PASI ≥1 and ≤ 5) or moderate-to-severe (PASI ≥ 10) plaque psoriasis;
5. Currently not using psoriasis medication and ≥ 2 plaques suitable for repeated biopsies and target lesion assessments. At least one of these lesions must be located on the extremities, preferably on the elbow or knee, with a minimal target lesion score between 6 and 9. Or, when currently using psoriasis medication and insufficient lesional skin is present, willing to discontinue treatment awaiting rescreening (see also exclusion criteria 3 for psoriatic patients);
6. Willing to give written informed consent and willing and able to comply with the study protocol;

And none of the following exclusion criteria at screening:

1. Having primarily erythrodermic, pustular or guttate psoriasis;
2. Having medication-induced psoriasis;
3. Having previously failed on anti-IL23 therapy;
4. Having received treatments for psoriasis within the following intervals prior to the start of the study:
5. < 2 weeks for topical treatment, e.g. retinoids, corticosteroids, vitamin D analogs
6. < 4 weeks for phototherapy, e.g. PUVA, PDT
7. < 4 weeks for non-biologic systemic treatment, e.g. retinoids, methotrexate, cyclosporine, fumaric acid esters
8. < 4 weeks for etanercept
9. < 8 weeks for adalimumab
10. < 3 months for anti-IL17, anti-IL12(/23) and anti-IL23 treatments
11. History or symptoms of any significant uncontrolled disease including (but not limited to), neurological, psychiatric, endocrine, cardiovascular, respiratory, gastrointestinal, hepatic, or renal disorder that may interfere with the study objectives, in the opinion of the Investigator, excluding psoriasis and conditions that are related to psoriasis;
12. History of immunological abnormality (e.g., immune suppression, severe allergy or anaphylaxis) that may interfere with study objectives, in the opinion of the Investigator;
13. Known infection requiring antibiotic therapy within the last 3 months prior to the study, including latent tuberculosis;
14. Systemic immunosuppressive or immunomodulatory treatment within 30 days prior to the study;
15. Body mass index (BMI) ≤ 18.0 or ≥ 40.0 kg/m^2^; during COVID-19 pandemic only ≤ 18.0 or > 33.0 kg/m^2^
16. Participation in an investigational drug study within 3 months prior to screening or more than 4 times a year;
17. Loss or donation of blood over 500 mL within three months prior to screening;
18. The use of any medication or vitamin/mineral/herbal/dietary supplement within less than 5 half-lives prior to study participation, if the Investigator judges that it may interfere with the study objectives. The use of paracetamol (up to 4 g/day) and is allowed;
19. History of alcohol consumption exceeding 5 standard drinks per day on average within 3 months of screening. Alcohol consumption will be prohibited from at least 12 hours preceding each study visit;
20. Any other condition that could interfere with the conduct of the study or the study objectives, in the opinion of the Investigator.
21. During COVID-19 pandemic: presence of high risk comorbidities: such as cardiovascular, respiratory or immune system disorders other than psoriasis and psoriasis arthritis

**Supplemental materials and methods**

Chemicals

HPLC-grade chloroform (Honeywell, Charlotte, North Carolina, United States), UPLC grade ethanol (Biosolve, Valkenswaard, the Netherlands), UPLC grade heptane (LiChorSolv, Merck, Darmstadt, Germany), UPLC grade isopropyl alcohol (Biosolve, Valkenswaard, the Netherlands), UPLC grade Methanol (Biosolve, Valkenswaard, the Netherlands) were used. The 0.25M potassium chloride solution was prepared by dissoling reagent grade potassium chloride (Sigma Aldrich, Saint-Louis, Missouri, USA) in ultrapure water from a Milli-Q Advantage A10 system (Merck, Darmstadt, Germany). Deuterated internal standard and ceramide standards were bought from Avanti Polar Lipids (Alabaster, Alabama, United States) or received from Evonik (Darmstadt, Germany). Polyphenylene sulfide tape (Nichiban, Tokyo, Japan) was used for tape stripping.

Tape stripping was performed after TEWL was determined and at the same location. To determine absolute ceramide abundances it is necessary to correct for the total amount of SC harvested by tape stripping. Therefore, the protein content on each tape strip was measured by near-infrared densitometry using a D-SquameScan 850A (Heiland Electronic, Wetzlar, Germany) to obtain the SquameScan value (sqv).^3^ Then, a 16 mm diameter circle was punched out from tapes 5, 6, 7, 8 from the center of the area where pressure was directly applied to the skin. The circles of tape were stored in in a 20 ml glass vial with chloroform:methanol (2:1) at <-40 °C prior to extraction. Tapes were extracted in three batches. Tapes were agitated by rotary shaker using an IKA S4000 at 120 rounds per minute at 40 °C for one hour. The solvent was collected and shaking repeated with 1 ml of chloroform:methanol:water (1:2:0.5), chloroform:methanol (1:1) and heptane:isopropylalcohol (1:1), sequentially, pooling the solvent of the 4 tapes per assessment together between each round of shaking. A liquid-liquid extraction was performed by the addition of 4 ml 0.25M KCl and the samples stored overnight at 4 °C to allow for phase separation. The organic layer was collected and the aqueous layer washed with 4 ml of chloroform. Organic layers were combined and filtered through 0.45 µm PVDF syringe filters (Grace, Deerfield IL, USA). After filtration, a aliquot of the samples were dried and reconstituted in 60 µl heptane:chloroform:methanol (95:2.5:2.5) with containing 10 µM CER[N(24deu)S(18)] for analysis by ultra-performance liquid chromatography–mass spectrometry on a Acquity UPLC H-class (Waters, Milford, MA, USA) with a PVA-silica column (5 μm particles, 100 × 2.1 mm i.d.) (YMC, Kyoto, Japan) hyphenated to a XEVO TQ-S mass spectrometer (Waters, Milford, MA, USA) operating with atmospheric pressure chemical ionization in positive mode. Samples were analyzed in three runs and each contained quality control samples from the same from combined stratum corneum extract pool and calibration curves. All patient samples at baseline showed sufficient responses and were used for relative analysis. Peak picking was performed using TargetLynx V4.1 (Waters, Milford, MA, USA). The monoisotopic response of ceramides corrected with the internal standard was used for corrections including adduct formation as determined from quality control samples and calibration curves, C-13 isotope abundance, difference in response based on molecular weight determined from calibration curves. This was further corrected using the cumulative sqv of tape 5, 6, 7 and 8 corrected with blanks to obtain the amount of ceramides in a quantitative manner. Two samples did not show any analytes. Full details and validation of the method is described by Boiten, *et al.* (2016).^4^

**Statistical analysis**

Analysis of baseline data, including pearson correlations, between groups was performed using Prism 9.0 (GraphPad, Software, Boston, USA). Profiles of controls, non-lesional psoriasis and lesional psoriasis were compared using Mixed Models for Repeated Measures and Multiple comparisons were conducted with Bonferroni’s test. Longitudinal analysis were performed in SAS 9.4 (SAS Institute Inc., Cary, NC, USA). Longitudinal analysis method with comparisons between Guselkumab and placebo are performed within a mixed effects model with treatment and time and treatment by time as fixed factors and subject as random factor SAS 9.4 (SAS Institute Inc., Cary, NC, USA). Any log-normal distributed data is log transformed before analysis, whereby zeros are treated as missing.  Analysis results are back transformed. Treatment graphs show the mean and 95% Confidence Interval and are reported by p-value. Common within-individual associations for paired measures were determined using Rmcorr in R Statistical Software (version 4.1.2, R Core Team 2021).^5^ Statistical significance is shown as: P<0.05: *, P<0.005: ** and P<0.005: ***.

**Table s2:** Changes in LSS in lesional psoriasis and changes in TEWL in lesional and non-lesional psoriasis after 0, 4 and 16 weeks of placebo (n=6) or guselkumab treatment (n=20). Values are presented as mean ± SD.

|  |  | **Placebo** | | | **Guselkumab** | | |
| --- | --- | --- | --- | --- | --- | --- | --- |
|  |  | week 0 | week 4 | week 16 | week 0 | week 4 | week 16 |
| LSS | | 6.8±1.2 | 6.5±2.0 | 6.3±2.6 | 7.3±1.0 | 4.6±1.5 | 1.2±1.6 |
| TEWL (g/m2/h) | Lesional | 42.6±17.1 | 35.6±7.9 | 40.2±22.0 | 36.7±16.3 | 29.7±12.7 | 16.3±8.9 |
|  | Non-lesional | 12.3±6.8 | 12.6±5.1 | 12.7±3.4 | 12.8±5.4 | 14.3±4.0 | 10.5±5.0 |

**Table s3:** The relative abundance of the ceramide profile in % and the absolute abundance in pmol/sqv of the lesional skin of psoriasis patients after 0, 4 and 16 weeks of placebo (n=6) or guselkumab treatment (n=20). The abundance of the ceramide profile of healthy controls (n=10) was determined during a single occasion and is shown as reference. Values are presented as mean ± SD.

|  | Controls | Placebo | | |  | Guselkumab |  |
| --- | --- | --- | --- | --- | --- | --- | --- |
|  |  | week 0 | week 4 | week 16 | week 0 | week 4 | week 16 |
| **Relative abundance (%)** | | | | | | | |
| NdS | 1.6±1.8 | 10.7±1.8 | 10.3±3.8 | 9.8±2.2 | 10.7±2.8 | 8.2±1.9 | 10.9±8.8 |
| NS | 10.4±3.1 | 28.3±3.4 | 28.0±3.9 | 30.1±2.7 | 30.0±3.8 | 25.3±5.4 | 14.3±5.7 |
| NP | 21.8±6.0 | 8.2±2.5 | 8.6±1.5 | 7.5±1.6 | 8.0±1.6 | 10.0±4.1 | 17.0±7.0 |
| NH | 11.6±2.8 | 7.8±1.7 | 8.8±2.5 | 8.3±2.4 | 7.9±2.0 | 10.6±3.8 | 10.9±1.7 |
| AdS | 6.1±1.6 | 3.6±1.1 | 3.6±0.9 | 3.1±0.5 | 3.5±1.2 | 3.3±1.6 | 5.5±1.8 |
| AS | 5.6±3.1 | 20.2±2.6 | 19.3±1.7 | 21.0±2.1 | 20.0±2.1 | 17.7±4.1 | 9.3±4.5 |
| AP | 12.9±3.1 | 5.9±1.6 | 6.1±1.8 | 5.1±1.0 | 5.5±1.5 | 8.0±3.0 | 12.0±3.7 |
| AH | 9.7±1.6 | 12.4±3.3 | 12.8±2.8 | 12.3±2.1 | 11.8±2.8 | 13.1±2.3 | 14.01±3.7 |
| EODS | 0.3±0.1 | 0.1±0.2 | 0.1±0.1 | 0.1±0.0 | 0.1±0.1 | 0.1±0.1 | 0.3±0.2 |
| EOS | 4.6±2.0 | 1.9±1.4 | 1.5±0.9 | 1.8±1.0 | 1.7±0.9 | 2.3±1.2 | 3.1±2.0 |
| EOP | 0.5±0.3 | 0.1±0.1 | 0.1±0.1 | 0.1±0.1 | 0.1±0.1 | 0.1±0.1 | 0.2±0.2 |
| EOH | 1.6±0.6 | 0.4±0.3 | 0.5±0.3 | 0.5±0.3 | 0.4±0.3 | 0.7±0.6 | 1.3±0.8 |
| OdS | 0.01±0.01 | 0.01±0.01 | 0.03±0.02 | 0.02±0.02 | 0.01±0.01 | 0.01±0.01 | 0.01±0.01 |
| OS | 0.6±0.4 | 0.2±0.1 | 0.2±0.1 | 0.2±0.1 | 0.2±0.2 | 0.2±0.1 | 0.7±0.6 |
| OP | 0.1±0.1 | 0.02±0.01 | 0.04±0.03 | 0.03±0.03 | 0.02±0.03 | 0.02±0.03 | 0.04±0.1 |
| OH | 0.5±0.3 | 0.1±0.1 | 0.1±0.1 | 0.1±0.1 | 0.1±0.1 | 0.2±0.2 | 0.6±0.4 |
| **Absolute abundance (pmol/sqv)** | | | | | | | |
| NdS | 22.81±8.72 | 4.56±3.00 | 10.65±2.96 | 8.08±4.24 | 8.92±5.53 | 5.77±2.96 | 19.86±4.24 |
| NS | 17.77±8.78 | 12.11±6.94 | 30.45±10.35 | 24.78±11.83 | 25.79±14.98 | 18.10±10.35 | 21.85±11.83 |
| NP | 40.44±31.27 | 3.97±3.97 | 14.05±5.26 | 5.88±2.34 | 7.31±4.42 | 6.35±5.26 | 34.98±2.34 |
| NH | 20.61±13.89 | 3.47±1.61 | 14.51±4.08 | 7.24±5.77 | 6.49±2.99 | 6.76±4.08 | 20.30±5.77 |
| AdS | 10.24±4.16 | 1.76±1.36 | 4.57±2.36 | 2.54±1.34 | 3.11±1.82 | 2.68±2.36 | 10.72±1.34 |
| AS | 9.19±6.06 | 8.92±4.85 | 21.54±7.12 | 17.23±8.27 | 17.66±10.15 | 13.09±7.12 | 14.14±8.27 |
| AP | 21.09±7.06 | 2.85±2.71 | 11.39±4.58 | 4.20±2.41 | 5.06±3.08 | 5.00±4.58 | 23.45±2.41 |
| AH | 15.73±4.74 | 5.36±2.50 | 17.72±5.02 | 10.32±6.57 | 9.86±4.99 | 9.39±5.02 | 24.83±6.57 |
| EODS | 0.63±0.55 | 0.04±0.08 | 0.20±0.04 | 0.05±0.03 | 0.05±0.04 | 0.04±0.04 | 0.49±0.03 |
| EOS | 7.73±4.34 | 1.05±1.10 | 3.31±1.00 | 1.57±1.47 | 1.52±0.97 | 1.32±1.00 | 5.43±1.47 |
| EOP | 1.06±1.14 | 0.06±0.06 | 0.18±0.06 | 0.11±0.12 | 0.07±0.05 | 0.10±0.06 | 0.44±0.12 |
| EOH | 2.69±1.73 | 0.24±0.25 | 1.19±0.29 | 0.40±0.44 | 0.35±0.23 | 0.41±0.29 | 2.44±0.44 |
| OdS | 0.03±0.03 | 0.004±0.003 | 0.02±0.02 | 0.02±0.02 | 0.01±0.02 | 0.01±0.02 | 0.01±0.02 |
| OS | 0.96±0.81 | 0.08±0.05 | 0.17±0.18 | 0.17±0.16 | 0.18±0.21 | 0.27±0.23 | 1.27±1.39 |
| OP | 0.20±0.17 | 0.01±0.01 | 0.04±0.04 | 0.03±0.04 | 0.02±0.03 | 0.03±0.05 | 0.08±0.16 |
| OH | 0.87±0.58 | 0.09±0.10 | 0.12±0.11 | 0.11±0.12 | 0.12±0.12 | 0.35±0.42 | 1.30±1.16 |


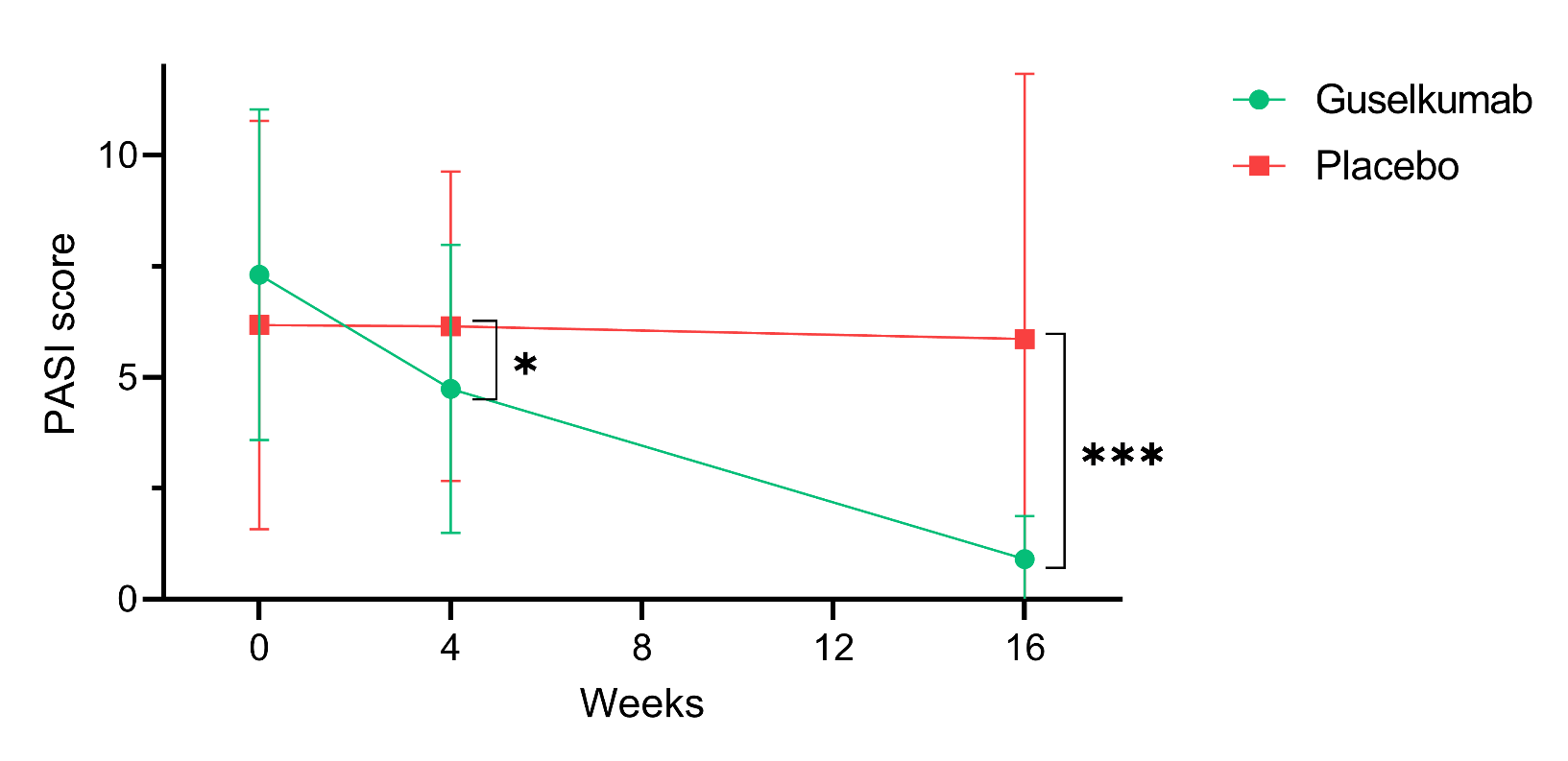


**Figure s3:** The Psoriasis Area and Severity Index (PASI) score over time in the guselkumab and placebo group. Graphs represent the mean and 95% Confidence Interval.


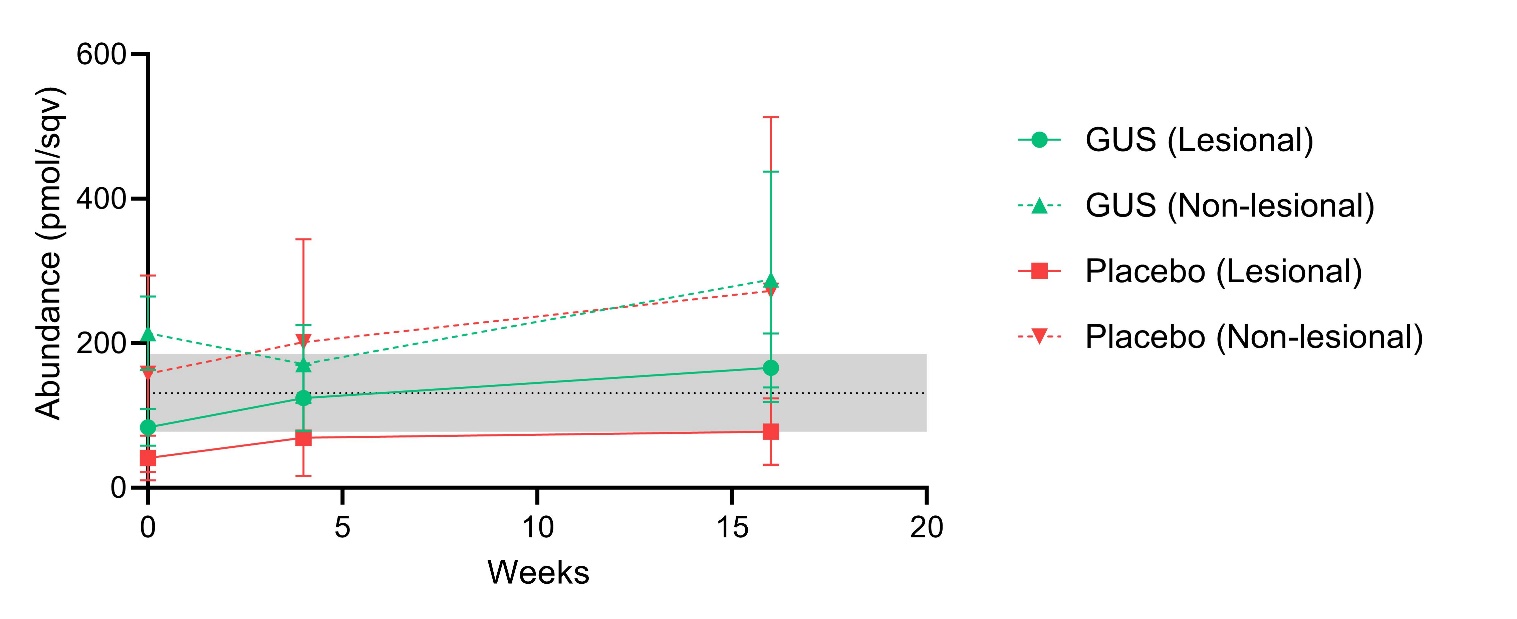


**Figure s4:** The absolute ceramide abundance during the treatment period indicates no evident effect of guselkumab treatment. The amount of ceramides is calculated by correcting the ceramide abundance with the SquameScan value (sqv). The sqv is proportional to the protein content removed with tape stripping and serves as an indicator for the total amount of SC on the tape strip. Graphs represent the mean and 95% Confidence Interval. The dotted black line and grey band represents the mean and 95% confidence interval of the control group at baseline.


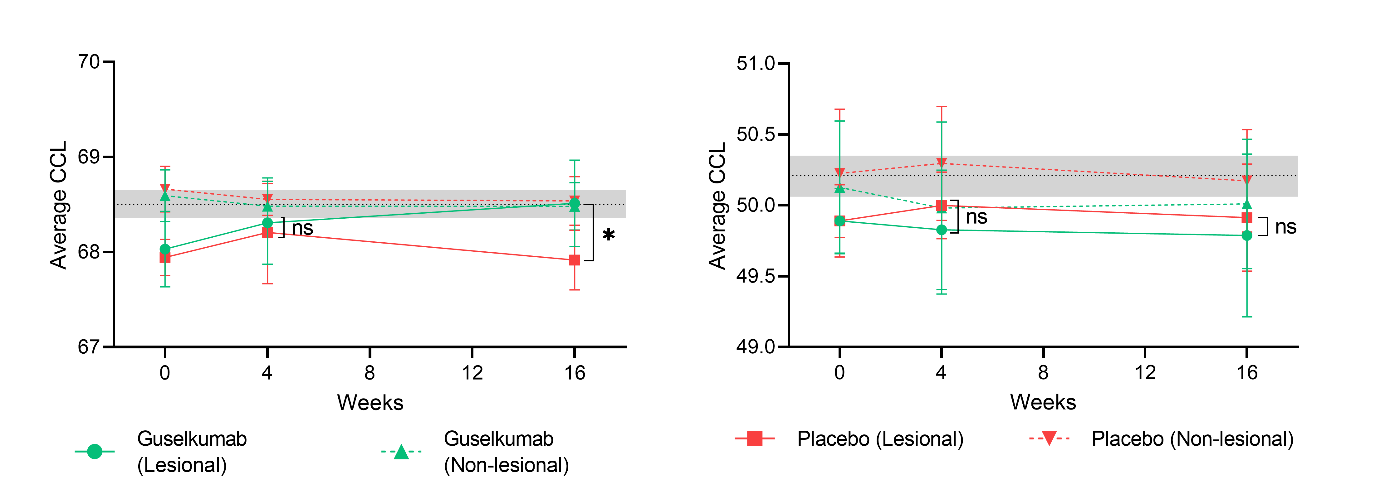


**Figure s5:** Ceramide chain length (CCL) over time during the treatment period. No significant differences are observed in the CCL of the Cer[EO] fraction (left) nor Cer[O] fraction (right) during treatment with guselkumab and placebo.

**Supplemental table s4:** The top 20 ceramides that contribute most for each principal component. These loadings are shown separately for both principal component 1 and principal component 2. The percentage variance explained by either component is shown in the header.

| **Principal component 1 (74.24%)** | | |  | **Principal component 2 (8.1%)** | | |
| --- | --- | --- | --- | --- | --- | --- |
| Rank | Ceramide | Loading |  | Rank | Ceramide | Loading |
| 1 | NS_c42 | 2.492177 |  | 1 | NP_c44 | 0.700184 |
| 2 | AS_c42 | 2.199156 |  | 2 | AH_c44 | -0.65358 |
| 3 | NS_c44 | 1.660657 |  | 3 | NP_c46 | 0.564729 |
| 4 | NP_c46 | -1.44715 |  | 4 | NS_c42 | 0.531482 |
| 5 | AS_c44 | 1.290758 |  | 5 | NP_c42 | 0.513692 |
| 6 | NP_c48 | -1.17746 |  | 6 | AH_c42 | -0.44367 |
| 7 | AS_c34 | 1.039784 |  | 7 | AH_c43 | -0.41148 |
| 8 | NP_c44 | -0.92791 |  | 8 | NS_c44 | 0.392519 |
| 9 | NS_c34 | 0.900425 |  | 9 | NP_c43 | 0.33858 |
| 10 | NS_c43 | 0.889957 |  | 10 | NP_c48 | 0.329555 |
| 11 | NS_c40 | 0.856169 |  | 11 | NdS_c42 | 0.303677 |
| 12 | AS_c43 | 0.81874 |  | 12 | AP_c46 | -0.29362 |
| 13 | AP_c46 | -0.81711 |  | 13 | AH_c45 | -0.25718 |
| 14 | NS_c41 | 0.766714 |  | 14 | AH_c46 | -0.24627 |
| 15 | NP_c47 | -0.73695 |  | 15 | NP_c45 | 0.235949 |
| 16 | NdS_c48 | -0.70814 |  | 16 | NH_c42 | -0.23554 |
| 17 | NP_c45 | -0.68668 |  | 17 | NS_c34 | 0.224409 |
| 18 | AH_c42 | 0.683911 |  | 18 | AS_c34 | -0.22321 |
| 19 | NdS_c42 | 0.651297 |  | 19 | NH_c41 | -0.21097 |
| 20 | NP_c43 | -0.57276 |  | 20 | AP_c45 | -0.20216 |

**Supplementary references**

1. Janssens, M. *et al.* Non-lesional skin in atopic eczema patients shows a change in lipid organization that correlates with a decreased barrier function. *Journal of Investigative Dermatology* **132**, S77 (2012).

2. Motta, S. *et al.* Ceramide composition of the psoriatic scale. *Biochim Biophys Acta* **1182**, 147–151 (1993).

3. Keurentjes, A. J., Jakasa, I. & Kezic, S. Research Techniques Made Simple: Stratum Corneum Tape Stripping. *Journal of Investigative Dermatology* **141**, 1129-1133.e1 (2021).

4. Boiten, W., Absalah, S., Vreeken, R., Bouwstra, J. & van Smeden, J. Quantitative analysis of ceramides using a novel lipidomics approach with three dimensional response modelling. *Biochimica et Biophysica Acta (BBA) - Molecular and Cell Biology of Lipids* **1861**, 1652–1661 (2016).

5. Bakdash, J. Z. & Marusich, L. R. Repeated measures correlation. *Front Psychol* **8**, 252904 (2017).
